# Spatial analysis of vaccine coverage on the first year of life in the northeast of Brazil

**DOI:** 10.1186/s12889-022-13589-9

**Published:** 2022-06-16

**Authors:** Nairmara Soares Pimentel Cunha, Sylvia Costa Lima Fahrat, Ricardo Alves de Olinda, Alfésio Luís Ferreira Braga, Carolina Luisa Alves Barbieri, Ysabely de Aguiar Pontes Pamplona, Lourdes Conceição Martins

**Affiliations:** 1 Catholic University of Santos (Universidade Católica de Santos – Programa de Pós- Graduação strictu senso em Saúde Coletiva), Av. Conselheiro Nebias, 300, sala 106; Santos, São Paulo, CEP: 11.015-002 Brazil; 2 Instituto da Criança, Hospital das Clínicas - Faculdade de Medicina da Universidade de São Paulo, São Paulo, Brazil; 3 Statistics Department, State University of Paraíba, Campina Grande, Brazil

**Keywords:** Vaccination, Spatial analysis, Global Moran’s Index, Local Moran’s Index, Mixed ecological study, Secondary data

## Abstract

**Background:**

Over time, vaccination has been consolidated as one of the most cost effective and successful public health interventions and a right of every human being. This study aimed to assess the spatial dynamics of the vaccine coverage (VC) rate of children aged < 1 year per municipality in the Brazilian Northeast at 2016 and 2017.

**Methods:**

This is a mixed-type ecological study that use a Public domain data Health Information. Vaccine doses were obtained from the Information System of the Brazilian National Immunization Program, and live births from the Brazilian Information System of Live Births of the Brazilian Unified Health System. Descriptive analysis of the coverage of all the vaccines for each year of the study was conducted, and Mann–Whitney U test was used to compare VC between the study years. Chi-squared test was used to evaluate the association between the years and VC, which was stratified into four ranges, very low, low, adequate, and high. Spatial distribution was analyzed according to both each study year and vaccine and presented as thematic maps. Spatial autocorrelation was analyzed using Moran’s Global and Local statistics.

**Results:**

Compared with 2017, 2016 showed better VC (*p* < 0.05), except for Bacillus Calmette–Guérin. In the spatial analysis of the studied vaccines, the Global Moran’s Index did not show any spatial autocorrelation (*p* > 0.05), but the Local Moran’s Index showed some municipalities, particularly the Sertão Paraibano region, with high VC, high similarity, and a positive influence on neighboring municipalities (*p* < 0.05). In contrast, most municipalities with low VC were concentrated in the Mata Paraibano region, negatively influencing their neighbors (*p* < 0.05).

**Conclusion:**

Uneven geographic regions and clusters of low VC for children aged < 1 year in the State of Paraíba were spatially visualized. Health policy makers and planners need to urgently devise and coordinate an action plan directed at each state’s regions to fulfill the vaccination calendar, thereby reversing the vulnerability of this age group, which is at a higher risk of diseases preventable by vaccination.

## Background

Vaccination is considered to be one of the most successful public health strategies, having saved countless lives and reduced the morbidity and mortality of several diseases, thereby allowing the complete and healthy development of children, making it the right of every human being. Vaccination is considered essential for any country’s future [1]. Over time, it has become globally established as one of the most cost effective health interventions [2].

Vaccination must be performed equitably and immunization services must be offered to all individuals regardless of geographical location, age, gender, socioeconomic or educational level, ethnicity, or occupation, according to the World Health Organization’s Global Vaccine Action Plan [2].

In Brazil, the National Immunization Program (PNI) has been highly successful and is used as a global reference. It aims to provide quality vaccination to the entire Brazilian population, particularly to all the children born in the national territory [3, 4] (Ministerio da Saúde (BR), 2017).

Vaccine coverage (VC) is an important performance indicator of PNI, characterized by 90% or more of the routine vaccines having their targets met in order to protect children against diseases preventable by vaccination, and also to protect the community as a whole, according to the Brazilian Program of Qualification of Health Surveillance Actions [5, 6].

The drop in VC in Brazil influences the infant morbidity and mortality increase and the regress of some diseases. Children are the segment of the population more susceptible to serious diseases, sequelae, and complications. Thus, fulfilling the child vaccination calendar is extremely important like as identifying regions with low VC is imperative for implementing prevention measures [6].

Spatial analysis is a fundamental method for detecting clusters whose space contributes to the evolution of the disease of identifying the low-coverage vaccination clusters areas, allowing for the identification of spatial and spatial–temporal clusters to recognize areas of greater vulnerability to health hazards [7]. Thus, spatial analysis plays an important role in identifying the geographic areas and population groups that are at risk of becoming ill or dying early due to the lack of vaccinations [8].

Therefore, the objective of this study is to use a geographic information system (GIS) and spatial analysis techniques to analyze the spatial dynamics of the VC rates and each vaccine’s spatial autocorrelation in children under the age of 1 year per municipality in the northeast Brazilian State in 2016 and 2017.

## Methods

This is a mixed-type ecological study with the municipality of residence as the analysis unit [9].

**Fig. 1:**
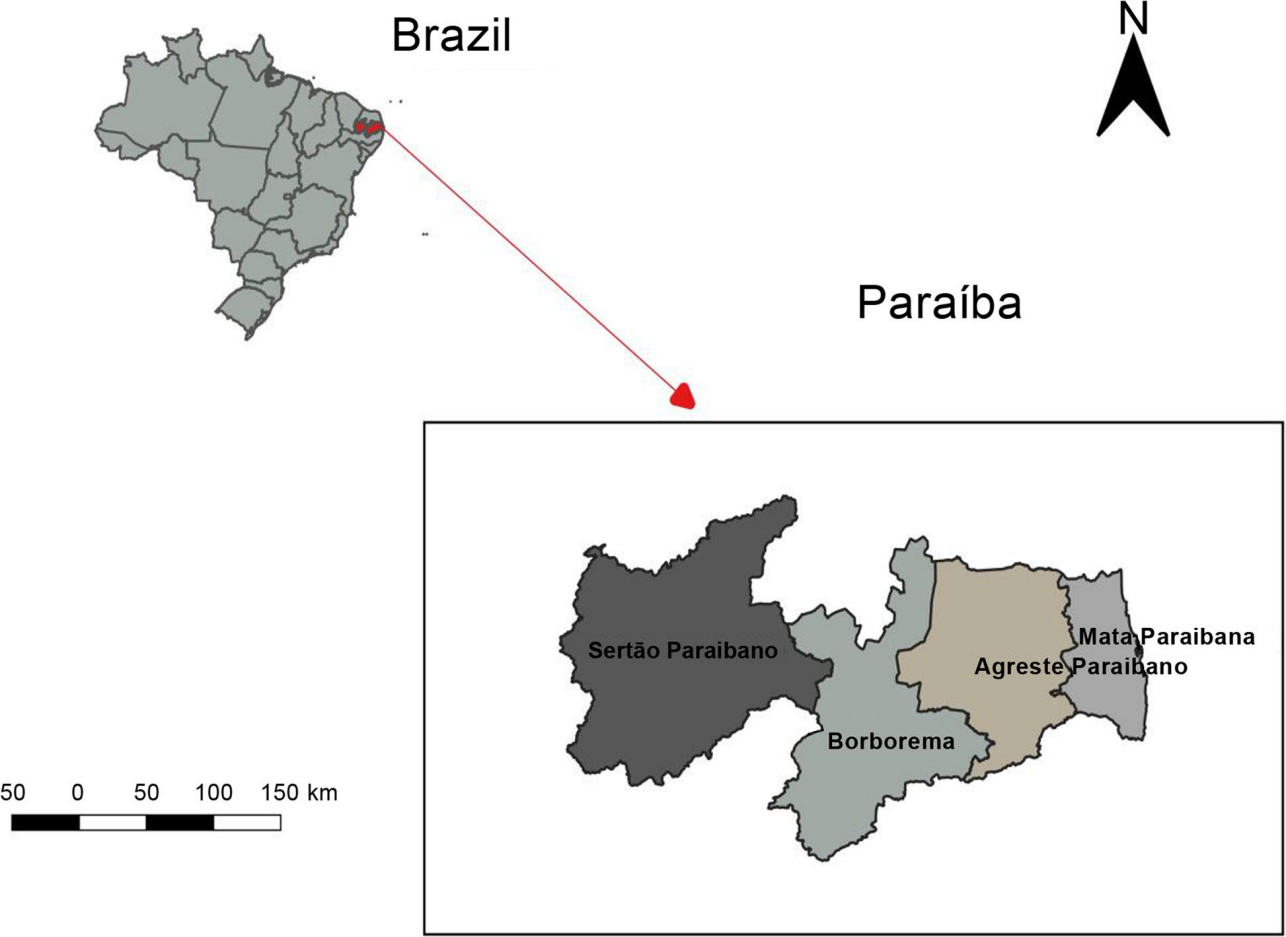
Map of Paraíba

The study used secondary, public-domain data from 2016 and 2017 of the 223 municipalities of the State of Paraíba (Fig. 1). The data were analyzed as aggregates without identifying the subjects to preserve the privacy and confidentiality of the information, according to the requirements presented by the resolution of the Brazilian National Health Council No. 466/2012, 510/2016, and 580/2018 regarding research with human beings, which emphasizes on dignity and respect for the research subjects [10–12].

This research is part of a broader project titled *Spatial Analysis of Children’s Vaccine Coverage and its Relationship with Socioeconomic and Health Characteristics in Brazil*, funded by the Bill and Melinda Gates Foundation and the Brazilian National Council for Scientific and Technological Development (CNPq).

Data were collected from the Department of Informatics of the Brazilian Unified Health System, which is responsible for collecting, processing, and publishing health information nationwide. Within this system, data from the Information System of the National Immunization Program (SI–PNI) and Information System on Live Births (SINASC) were used.

The applied doses of each vaccine for calculating VC were obtained from SI-PNI and data on live births were obtained from SINASC, both collected by year and municipality of residence.

The cartographic base of the municipalities’ digital mapping was obtained from the Brazilian Institute of Geography and Statistics, with geographic projection from the SIRGAS 2000 geodetic reference system [13].

VC was calculated by antigen administered to children aged under 1 year as a fraction using either the number of doses applied (for single-dose vaccines) or the number of last doses applied (for multiple-dose vaccines) as the numerator, and the live-birth data per year and municipality as the denominator.

The vaccines considered in the calculation of VC are part of the Brazilian National Vaccination Calendar for children aged under 1 year: Bacillus Calmette–Guérin (BCG) at birth; vaccine against Hepatitis B (HepB) at birth and at 2, 4, and 6 months; vaccine against meningococcus type C (MnCc) at 3 and 7 months; vaccine against Diphtheria–Tetanus–Pertussis (DTP), vaccine against *Haemophilus influenzae* type B (HiB), vaccine against poliomyelitis (polio), Rotavirus (rota), and Pneumococcus (pneumo), all at 2, 4, and 6 months.

The following formula was used to calculate the coverage of each vaccine:

$$VC=\frac{Doses\;of\;each\;vaccine\;given\;in\;that\;municipality\;and\;year}{Live\;births\;in\;that\;municipality\;and\;year}\ast100$$

VC was stratified into four categories: very low (from 0 to 50%), low (from 50% to the target), adequate (from the target to 120%), and high (≥ 120%) [5].

The PNI agreed upon the target of 90% of the population below the age of 1 year to be immunized with the BCG and rota vaccines, and 95% with the DTP, HepB, polio, pneumo, and MnCc vaccines. Attaining these coverage rates is considered to be adequate and signifies that the target has been met.

A descriptive analysis of all the study variables was performed. Quantitative variables were analyzed in terms of their central tendency and dispersion values. Qualitative variables were analyzed in terms of their absolute and relative values, and Chi-squared test was used to evaluate the association between the years and the categorized VCs. SPSS version 24.0 (IBM Corporation, Armonk, NY, USA) was used for the statistical analysis of the study data.

For analyzing the spatial dynamics, thematic maps were built from the calculation of VC for each vaccine by municipality and year, using thematic cartography of Geographic Information System (GIS).

The maps were built in QGIS 3.10 (QGIS Development Group), a free and open-source GIS that allows for the analysis of georeferenced data. According to the definitions of thematic representation, two dark colors were defined: red, meaning within the established target, and purple, meaning above the target; and two light colors, which translate into below target and far below target.

In spatial analysis, it is necessary to understand two fundamental principles: spatial dependence and spatial autocorrelation. Spatial dependence signifies that most natural or social events demonstrate a relationship between each other, while spatial autocorrelation is the measurement of that relationship via indicators [14].

Therefore, autocorrelation is determined by evaluating the similarity between a location and an attribute, and it is necessary to implement a matrix of weights. One of the most employed autocorrelation measures is Moran’s Binary Spatial Weights Matrix, which considers the spatial autocorrelation to be positive when the location and attribute are similar, negative when they are not similar, and close to 0 when the attribute values are random and independent in space [15].

This study used the neighborhood matrix “w” of first order, which signified that municipalities sharing a common physical border are considered as neighbors. The connected regions are believed to interact more than unconnected ones, and these connections are represented by matrix, wherein a value of 1 signifies a common border and 0 signifies no border.

The spatial autocorrelation analysis was initially performed using the Global Moran’s Index (*I*), which identified the State of Paraíba as a unique study area and allowed a general measurement of the spatial association. However, as it does not allow for the detailed analysis of spatial patterns, and thus, the identification of the spatial correlations between the municipalities, the Local Index of Spatial Association (LISA) was used to identify areas with values of similar attribute (clusters) and they were visualized through Moran’s Map.

In the Moran’s Map, “high-high” was identified in red, which indicated a municipality with high VC and a positive influence on its neighbors (i.e., neighbors with high VC); “low-low” was identified in blue and indicated a municipality with low VC and a negative influence on its neighbors (i.e., municipalities with low VC); “high-low” was identified in green and indicated the municipalities with high VC surrounded by low VC municipalities; “low–high,” indicated in light blue included the municipalities with low VC surrounded with cities with high VC; and nonsignificant was indicated in white and included municipalities with no statistically significant spatial autocorrelation (*p* ≤ 0.05).

The Global Moran’s Index, LISA, and Moran’s Map analyses were performed using the free and open-source software R Studio (R Development Core Team, 2019) with the spatial autocorrelation and LISA tools. The adopted significance level was 5%.

## Results

In this study, the VC data, one of the PNI performance indicators, were analyzed for 2016 and 2017 in the 223 municipalities of the State of Paraíba (Table 1). In 2016, Paraiba’s total CV was 50.10% and in 2017 it was 70.08%.

**Table 1:** Descriptive vaccine coverage analysis in children aged < 1 year in Paraíba region (2016 and 2017)

	**2016**		**2017**		***p* value**
	**N**	**%**	**N**	**%**	
Bacillus Calmette–Guérin					
Very low (0–50)	110	49.3	104	46.6	
Low (50–90)	61	27.4	66	29.6	0.942
Adequate (90–120)	34	15.2	34	15.2	
High (> 120)	18	8.1	19	8.5	
Hepatitis B					
Very low (0–50)	7	3.1	5	2.2	
Low (50–95)	106	47.5	136	61.0	0.004
Adequate (95–120)	77	34.5	69	30.9	
High (> 120)	33	14.8	13	5.8	
*Haemophilus influenzae* type B					
Very low (0–50)	8	3.6	5	2.2	
Low (50–95)	105	47.1	136	61.0	0.004
Adequate (95–120)	78	35.0	69	30.9	
High (> 120)	32	14.3	13	5.8	
Diphtheria–Tetanus–Pertussis					
Very low (0–50)	8	3.6	5	2.2	
Low (50–95)	105	47.1	136	61.0	0.004
Adequate (95–120)	78	35.0	69	30.9	
High (> 120)	32	14.3	13	5.8	
Poliomyelitis					
Very low (0–50)	9	4.0	2	0.9	
Low (50–95)	105	47.1	137	61.4	0.001
Adequate (95–120)	66	29.6	67	30.0	
High (> 120)	43	19.3	17	7.6	
Rotavirus					
Very low (0–50)	7	3.1	4	1.8	
Low (50–90)	68	30.5	95	42.6	0.004
Adequate (90–120)	121	54.3	114	51.1	
High (> 120)	27	12.1	10	4.5	
Pneumococcus					
Very low (0–50)	4	1.8	3	1.3	
Low (50–95)	77	34.5	107	48.0	0.008
Adequate (95–120)	104	46.6	94	42.2	
High (> 120)	38	17.0	19	8.5	
Meningococcus					
Very low (0–50)	7	3.1	3	1.3	
Low (50–95)	91	40.8	125	56.1	0.002
Adequate (95–120)	95	42.6	82	36.8	
High (> 120)	30	13.5	13	5.8	

Spatial analysis using the spatial autocorrelation indicator Global Moran’s Index found no statistically significant spatial autocorrelation in the two years (Table 2).

**Table 2:** Global Moran’s Index

**Covariate** **Vaccine Coverage**	**Year**	**Global**	
		**Moran’s Index**	***p* value**
Bacillus Calmette–Guérin	2016	0.020	0.2806
	2017	0.0580	0.0695
Hepatitis B	2016	0.0424	0.1345
	2017	0.0618	0.0589
*Haemophilus influenzae* type B	2016	0.0416	0.1387
	2017	0.0618	0.0589
Diphtheria–tetanus–pertussis	2016	0.0416	0.1387
	2017	0.0636	0.0542
Poliomyelitis	2016	0.0636	0.0530
	2017	− 0.0182	0.6291
Rotavirus	2016	0.0599	0.0639
	2017	0.0339	0.1787
Pneumococcus	2016	− 0.0066	0.5204
	2017	0.0155	0.3179
Meningococcus	2016	0.0280	0.2211
	2017	0.0347	0.1772

When calculating the Spatial Association Index, LISA, also known as the Local Moran’s Index, it is possible to perform the local spatial autocorrelation, and thus, visualize the municipalities with similarities and form clusters of high and low VC, which consequently positively or negatively influence their neighbors.

**Fig. 2:**
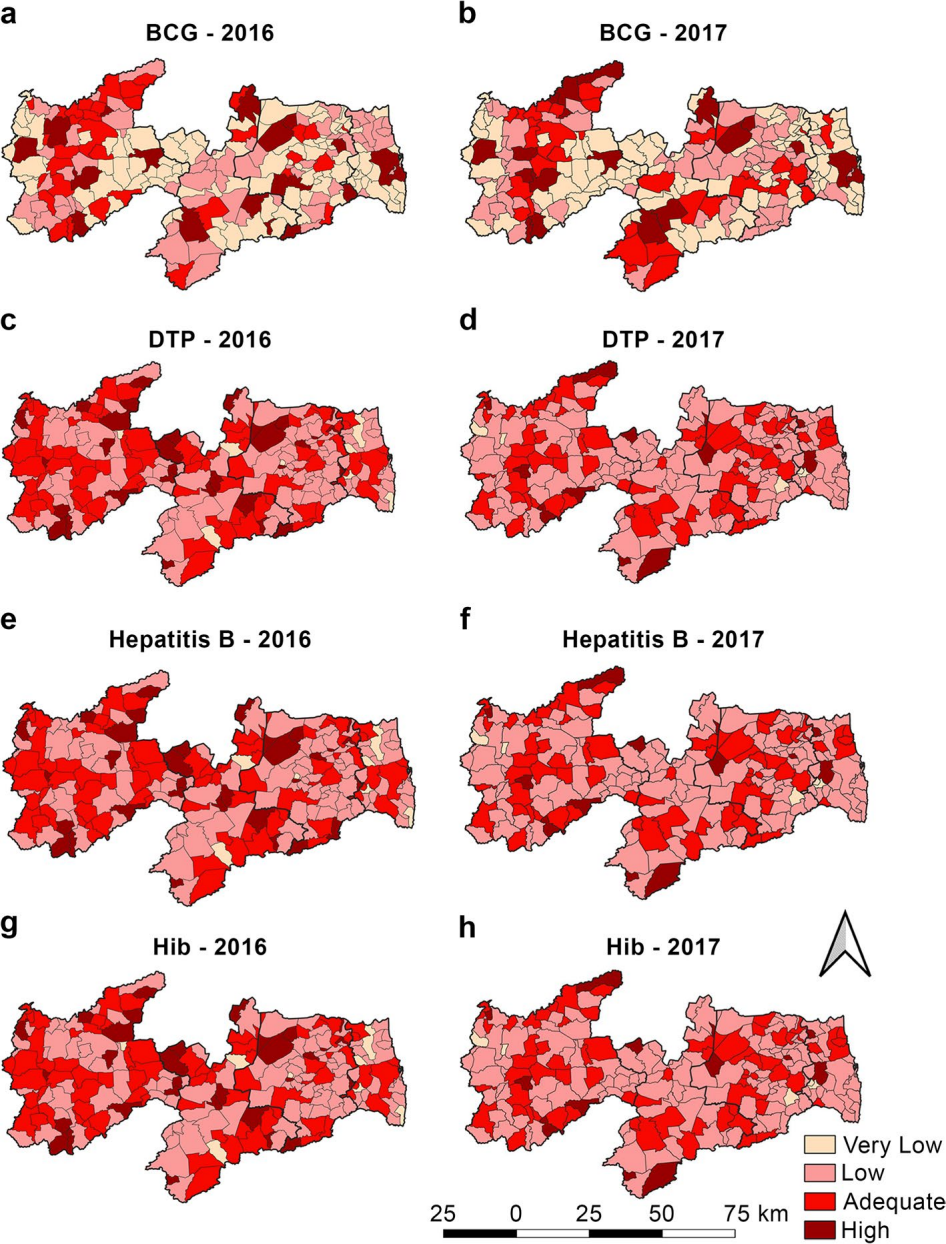
Vaccine coverage for *Bacillus Calmette*–Guérin (BCG), Diphtheria–Tetanus–Pertussis (DTP), hepatitis B, and *Haemophilus influenzae* type B (HiB) in 2016 and 2017, State of Paraíba, Brazil

**Fig. 3:**
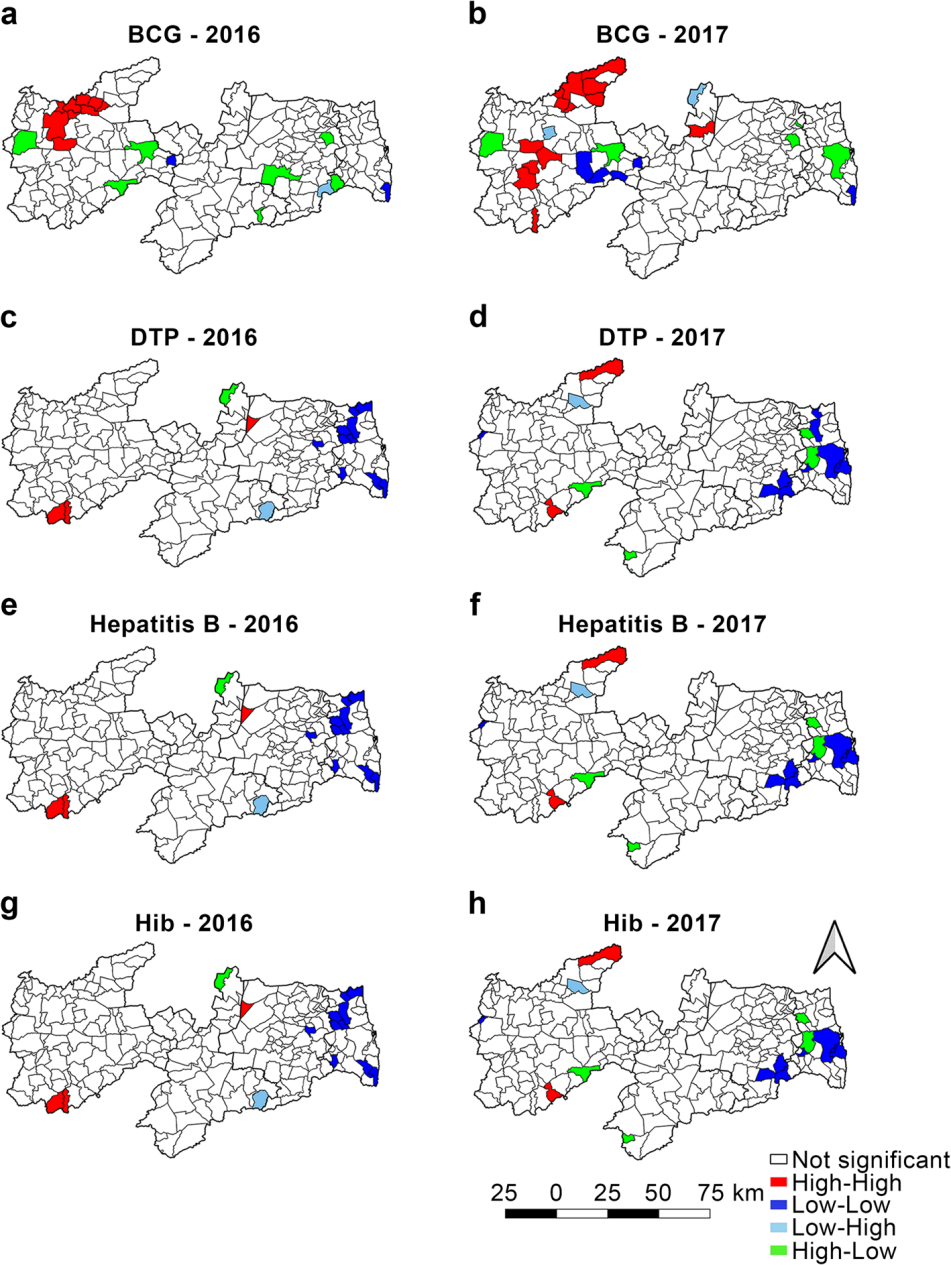
Moran’s Map of the vaccines given to children under 1 year of age. Paraíba, 2016–2017

**Fig. 4:**
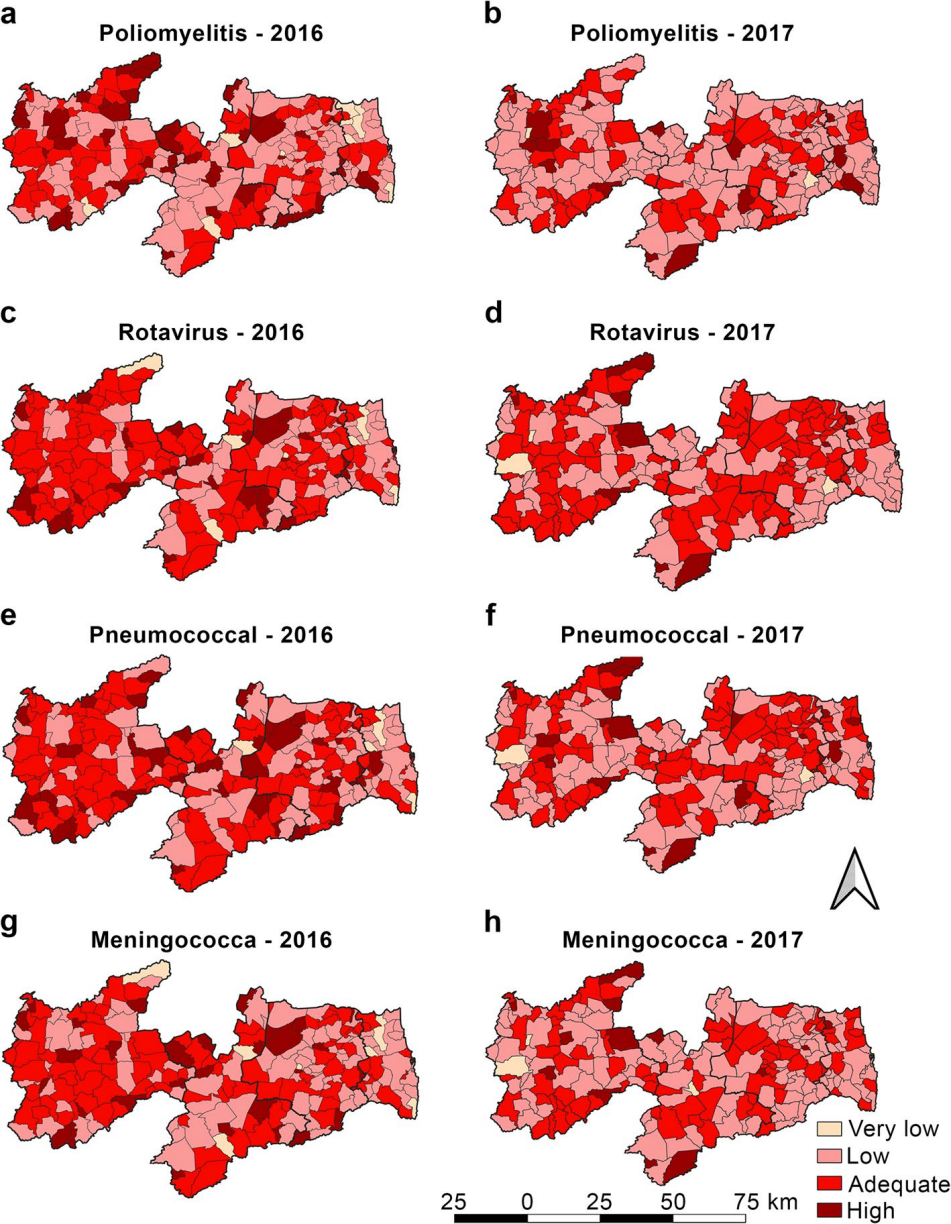
Vaccine coverage for poliomyelitis, rotavirus, pneumococcus, and meningococcus in 2016 and 2017, State of Paraíba, Brazil

**Fig. 5:**
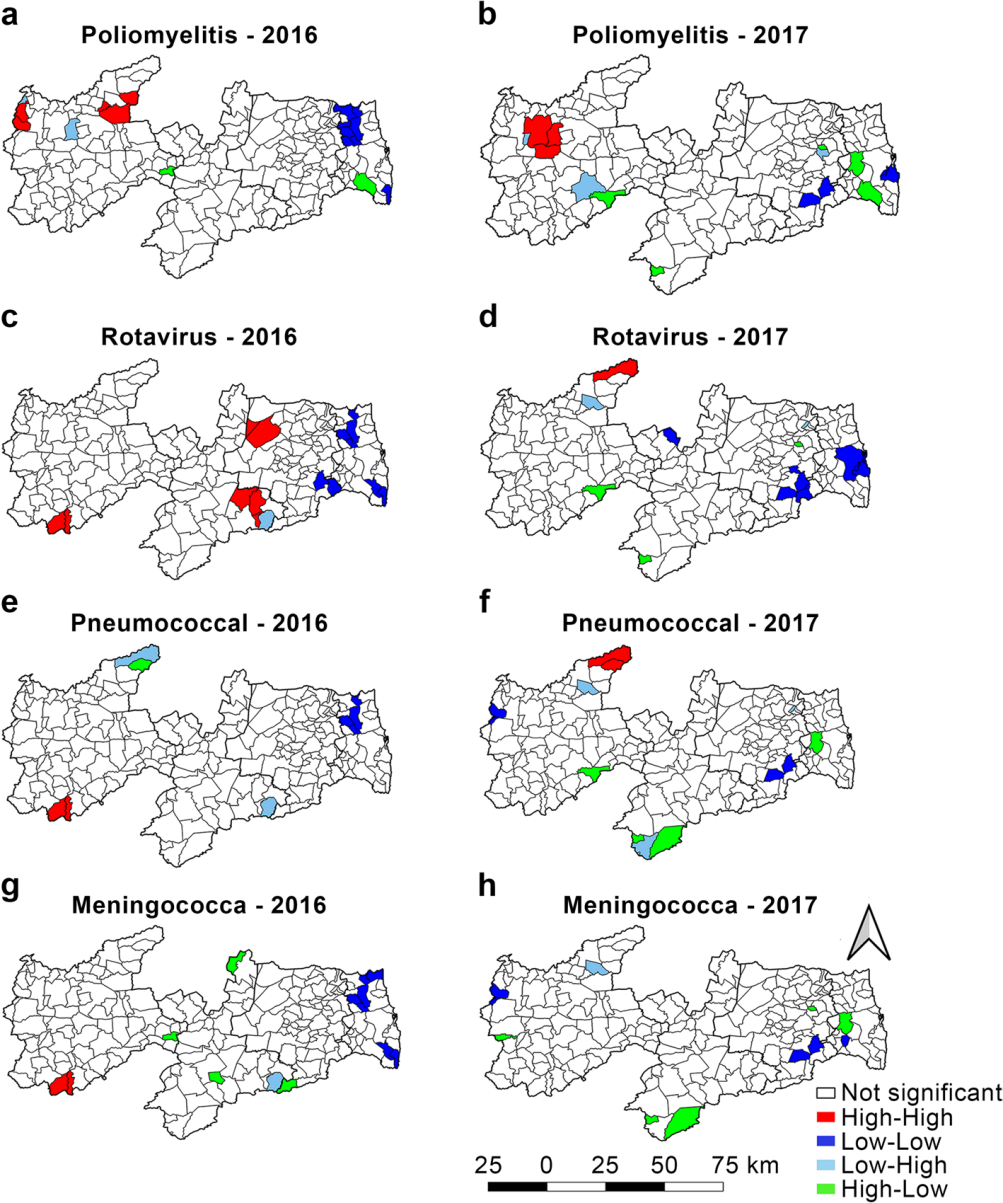
Moran’s Map of the vaccines given to children aged under 1 year. Paraíba, 2016–2017

Figures 2 and 4 show the spatial distribution of VC and Figs. 3 and 5 show the spatial autocorrelations of all the vaccines administered to children aged under 1 year.

Only 15.2% (Table 1) of the municipalities showed an adequate VC for BCG in the two years, and the Sertão Paraibano mesoregion comprised the largest number of municipalities with adequate BCG VC in 2016 (Fig. 2a). In the spatial analysis of BCG VC in 2016 (Fig. 3a), a cluster of municipalities were observed in the Sertão Paraibano mesoregion with high similarity, high VC, and positive influence on their neighbors. In 2017 (Fig. 3b), some agglomerations of Sertão Paraibano municipalities were seen, including one cluster toward the north and another in the center of the region with high VC and high similarity, and a third cluster toward the east with low VC and a negative influence on their neighbors.

Figure 2 shows the set formed by the DTP, HepB, and HiB vaccines, which will be discussed simultaneously as it is administered together as a pentavalent vaccine. In 2016, the Sertão Paraibano showed the most municipalities with adequate VC forming clusters for these vaccines (Fig. 2c, e, and g). By contrast, in 2017 (Fig. 2d, f, and h), a cluster of municipalities with adequate DTP, HepB, and HiB VC was seen in the mesoregions Sertão Paraibano, Agreste Paraibano and Borborema. However, only 30.9% of the municipalities showed adequate VC for DTP, HepB, and HiB and 63.2% showed VC below the target set forth by Brazilian National Immunization Program (Table 1).

The spatial analysis for 2016 (Fig. 3c, e, g) showed only 14 municipalities with statistical significance (*p* < 0.05). A cluster of municipalities was seen toward the north of the Mata Paraibano region, as well as two municipalities on the coast, all negatively influencing their neighbors. This region included João Pessoa, the state capital. Only two municipalities in the Sertão Paraibano region showed high DTP, HepB, and HiB VC, with a positive influence on their neighbors. In 2017, the spatial distribution pattern (Fig. 3d, f, h) was the same for DTP, HepB, and HiB, except for DTP in one municipality of the Mata Paraibano, which showed low VC and a negative influence on its neighbors.

Figure 4 shows that the polio, rota, and pneumo vaccines had completely different spatial patterns in the two years analyzed. These vaccines must be simultaneously administered with the application of DTP, HiB, and HepB, according to the child vaccination calendar recommended by the Brazilian PNI.

In 2016 (Table 1), adequate VC for polio was seen in only 29.6% of the Paraíba municipalities, and most municipalities presented VC below the PNI target. A larger cluster of adequate polio VC was observed in the Sertão Paraibano region (Fig. 4a). In the spatial distribution (Fig. 5a), few municipalities showed a positive spatial autocorrelation (*p* < 0.05); these included a cluster in the east and west of Sertão Paraibano region with high VC and a positive influence on their neighbors. In the north of the Mata Paraibana region, a cluster of municipalities with low polio VC and a negative influence on their neighbors was seen.

In 2017, (Table 1) 62.3% of the Paraíba municipalities were below the PNI target for polio VC. Only 30% of the municipalities showed adequate polio VC in the Sertão Paraibano region (Fig. 4b). In the Mata Paraibana mesoregion, most municipalities were in the low polio VC. Spatial analysis (Fig. 5b) showed a cluster of municipalities in the center of the Sertão Paraibano region with positive influence on their neighbors. João Pessoa state capital city showed a lower VC and a negative influence on its neighbors.

In 2016, rota VC (Fig. 4c) showed a cluster of municipalities in the Sertão Paraibano region with adequate VC. In the Mata Paraibana region was observed an adequate VC but with dispersed pattern. In the spatial analysis for rota (Fig. 5c), a few municipalities showed spatial autocorrelation (*p* < 0.05), with high VC and a positive influence on their neighbors. A cluster of municipalities can also be seen in the northwest and the south of the Mata Paraibana region, and in the southeast of the Agreste Paraibano region, with low rota VC and a negative influence on their neighbors.

In 2017, (Table 1), 51.1% of the municipalities showed adequate rota VC (Fig. 4d). In the spatial analysis for rota VC (Fig. 5d), the municipalities with spatial autocorrelation (*p* < 0.05) included a high VC cluster toward the north of Sertão Paraibano but not influence its neighbors. A few municipalities in the Mata Paraibana and Agreste regions showed low rota VC and a negative influence on their neighbors.

For pneumo in 2016 the Sertão Paraibano was the region with adequate VC (Fig. 4e), although in the Mata Paraibana region, a cluster of low VC (Table 1). Spatial analysis (Fig. 5e) showed that only eight municipalities in the state demonstrated a positive spatial autocorrelation (*p* < 0.05), of these, only two, located in the Sertão Paraibano region, showed high pneumo VC and a positive influence on their neighbors. A cluster of municipalities in the Mata Paraibana region was observed with low pneumo VC and a negative influence on their neighbors.

In the 2017 VC data for pneumo (Fig. 4f), clusters of municipalities with high pneumo VC can be seen in the regions of Sertão Paraibano, central Borborema, the Agreste Paraibano, and the north coast of the Mata Paraibana (Table 1). In the spatial analysis (Fig. 5f), 13 municipalities showed statistical significance (*p* < 0.05), but only two municipalities showed high VC and a positive influence on their neighbors, both in the north of the Sertão Paraibano region. It was also seen that two municipalities, one in the Agreste Paraibano region and another in the Sertão Paraibano region, showed low pneumo VC and a negative influence on their neighbors.

In the 2016 MnCc VC (Fig. 4g), clusters of municipalities with adequate VC were observed in the Sertão Paraibano and Borborema regions, 42.6% municipalities showed adequate MnCc VC and 43.9% were below the recommended target (Table 1). In the spatial distribution (Fig. 5g), a few municipalities with positive spatial autocorrelation (*p* < 0.05) were seen and only two municipalities with high MnCc VC, high similarity, and a positive influence on their neighbors both in the Sertão Paraibano region. The Mata Paraibana showed spatial autocorrelation (*p* < 0.05) with low MnCc VC and a negative influence on their neighbors.

In the 2017 MnCc VC (Fig. 4h), whole municipalities presented low MnCc VC (Table 1). Spatial analysis (Fig. 5h) showed that a few municipalities presented positive spatial autocorrelation (*p* < 0.05), but none had high MnCc VC with a positive influence on its neighbors.

## Discussion

In 2016 and 2017, when evaluating VC, stratified as very low, low, adequate, and high, a considerable number of municipalities with low or very low VC according to the PNI targets were observed for all the vaccines analyzed in this study. In particular, for BCG, 49.3% and 46.7% of the municipalities had VC < 50% in 2016 and 2017, respectively, and only 15.2% of the municipalities had adequate VC in both the years.

The VCs a that are administered in the same period according to the Brazilian National Vaccination Calendar for children aged under 1 year (DTP, HiB, HepB, polio, rota, and pneumo) showed a difference among municipalities in the study period. This further signified that there is a loss of vaccination opportunities and the moment of making the indicated vaccines for this age range available is being missed in the vaccination rooms of all the municipalities of Paraíba, which goes against the guidelines of both PNI and the World Health Organization. This confirms the need to identify factors that might be influencing this loss of vaccination opportunities. These results agree with those by Barata et al. [16], Arroyo et al. [17], and Ferreira et al. [18].

When analyzing the spatial distribution of all the evaluated vaccines, a large number of municipalities with no statistical significance were found, further corroborating that VC in the Paraíba region is far below the targets set by PNI. From all the results of the spatial analysis, it can be inferred that for most vaccines, the Sertão Paraibano region had the most municipalities with adequate VC. Additionally, in the spatial autocorrelation, that region showed the largest number of spatial clusters with high VC and high similarity. By contrast, the Mata Paraibana region, where the state capital is located, presented clusters of low VC and a negative influence on neighboring municipalities.

The low VC found in 2016 and 2017 have been confirmed by other studies, some of whom analyzed the nationwide VC [5, 17, 19, 20], while others assessed VC at the state level [21–23].

The present study found a heterogeneous distribution of VC among the municipalities, which is corroborated by the study of Barata et al. [16], which evidenced the inequality of VC among Brazil’s 27 state capitals and showed clusters of low VC within those cities regarding the vaccine recommendations until 18 months of age. In the study by Arroyo et al. [17], which analyzed areas with decreased VC in Brazil from 2006 to 2016, it was shown that BCG and polio had the lowest VC rates in 2016. The study also demonstrated the heterogeneous spatial distribution of the drop in VC among the country’s various regions. For BCG, the study’s 2016 results agreed with those of the present study, which reported VC to be below target in both the years. By contrast, the 2016 polio VC data in the above study are not similar with that of the present study, although the 2017 VC are similar.

The data found in this study for the BCG, DTP, and polio VC in the Paraíba, showing a heterogeneous VC distribution among the state municipalities, are corroborated by the findings of Khan, Shil, and Prakash [24] in India, and those for BCG and DTP by the findings of Vyas, Kim, and Adams [25] in Bangladesh.

Considering the state’s mesoregions, it was observed that the Sertão Paraibano region had the largest number of municipalities showed high VC and high similarity with their neighbors. In the Mata Paraibana region, where the state capital is located, we have been observed a low VC clusters negatively influencing the neighboring municipalities.

Khan, Shil, and Prakash [24] also stated that spatial analyses at subnational levels are important because they allow spatial disparities in health to be assessed, identifying low VC areas. According to Joy et al. [26], identifying areas with low VC is important to avoid epidemics of diseases preventable by vaccination and the implementation of immunization strategies.

A study conducted by Yourkavitch et al. [27] concluded that spatial analysis is important for understanding the distribution of health indicators, identifying low-performing regions, and addressing inequalities in health. Brearley et al. [28] stated that understanding the peculiarities of low-performing geographical cluster and the factors that promote low VC are essential for health policy makers and planners who aim to meet the VC targets.

It is also important to mention that few Brazilian studies have assessed VC by municipalities using cartographic resources. Other than Barata et al. [16], Arroyo et al. [17], Martins et al. [29] and Barbieri et al. [30], no studies could be found that analyzed VC in children aged less than 1 year using Moran’s Index to measure spatial autocorrelation.

It is important to highlight the possibilities presented by this study, including the formation of a database that allowed for a more accurate VC calculation and visualization of the spatial distribution of “vaccination clusters” through spatial analysis. Additionally, assessing VC through spatial analysis is an innovative approach in health care, as few Brazilian and international studies have addressed this topic that is relevant to the worldwide public health.

The inherent limitations of ecological studies that were present in this study were corrected by using spatial analysis techniques. The possibility of errors in the demographic database, particularly when using the estimated population of children aged less than 1 year, especially in years between censuses [31], was corrected by using SINASC data. To resolve possible inconsistencies in the data regarding the doses administered in vaccination facilities, VC for each vaccine was calculated by extracting the SI–PNI data by the place of residence.

## Conclusion

This study performed the spatial visualization of geographical areas and clusters of low VC in children aged under 1 year among the municipalities of the State of Paraíba, Brazil. The existence of these areas demonstrates an urgent need to devise and coordinate an action plan by the state and municipal public policy makers and health planners directed toward each region. The incomplete vaccination of children aged under 1 year evidences the need of greater efforts to ensure the completion of the vaccine calendar.

## Data Availability

We declare that the data used are from the public domain health (link: https://datasus.saude.gov.br) and were obtained according to the criteria of good research practice and ethical precepts.

## Abbreviations

GIS: Geographic information system
LISA: Local Index of Spatial Association
VC: Vaccine coverage
BCG: Bacillus Calmette–Guérin
DTP: Diphtheria–Tetanus–Pertussis
HepB: Hepatitis B
HiB: *Haemophilus influenzae* Type B
MnCc: Meningococcus type C
PNI: National Immunization Program
SINASC: Information System on Live Births

## References

[1] World Health Organization (WHO) (2013). Global vaccine action plan 2011–2020.

[2] World Health Organization (WHO) (2018). Assessment report of the Global Vaccine Action Plan - Strategic Advisory Group of Experts on Immunization.

[3] Ministério da Saúde (BR) (2017). Secretaria de Vigilância em Saude. Programa Nacional de Imunizações - vacinação.

[4] Ministério da Saúde (BR) (2003). Programa Nacional de Imunização 30 anos.

[5] Braz RM, Domingues CMAS, Teixeira AM da S, Luna EJ de A (2016). Classificação de risco de transmissão de doenças imunopreveníveis a partir de indicadores de coberturas vacinais nos municípios brasileiros. Epidemiol Serv Saúde.

[6] BRASIL. Ministério da Saúde. Secretaria de Vigilância em Saúde. Departamento de Análise em Saúde e Vigilância de Doenças não Transmissíveis. Avaliação dos indicadores de desempenho da vacinação do Programa Nacional de Imunizações e os desafios para elevar as coberturas vacinais no Brasil. Saúde Brasil 2019: uma análise da situação de saúde com enfoque nas doenças imunopreveníveis e na imunização. Brasília: Ministério da Saúde; 2019. p. 369–403. cap. 17.

[7] Chiaravalloti-Neto F (2017). O geoprocessamento e saúde pública. Arq Cienc Saude.

[8] Ministério da Saúde (BR), Secretaria de Vigilância em Saúde, Fundação Oswaldo Cruz (2006). Abordagens espaciais na saúde pública: Série Capacitação e Atualização em Geoprocessamento em Saúde.

[9] Morgenstern H (1995). Ecologic studies in epidemiology: concepts, principles, and methods. Annu Rev Public Health.

[10] Guerriero ICZ (2016). Resolução n^o^ 510 de 7 de abril de 2016 que trata das especificidades éticas das pesquisas nas ciências humanas e sociais e de outras que utilizam metodologias próprias dessas áreas. Cien Saude Colet.

[11] CONEP. Conselho Nacional de Saúde.RESOLUÇÃO No 580, DE 22 DE MARÇO DE 2018. Disponível em: https://conselho.saude.gov.br/resolucoes/2018/Reso580.pdf. Accessed on 20 Aug 2022.

[12] CONEP. Conselho Nacional de Saúde.RESOLUÇÃO No 466, DE 12 DE DEZEMBRO DE 2012. Disponível em: https://bvsms.saude.gov.br/bvs/saudelegis/cns/2013/res0466_12_12_2012.html. Accessed on 20 Aug 2022.

[13] Instituto Brasileiro de Geografia e Estatística (IBGE) (2016). Mapas: bases e referenciais: bases cartográficas: malhas digitais: municipal.

[14] Câmara G, Carvalho MS, Cruz OG, Correa V, Druck S, Carvalho MS, Câmara G, Monteiro AVM (2004). Análise espacial de áreas. Análise espacial de dados geográficos.

[15] Sistemas e ciência da informação geográfica [recurso eletrônico]/ Paul A. Longley... [et al.]; [tradução: André Schneider... et al.]; revisão técnica: Heinrich Hasenack, Eliseu José Weber. – 3. ed. – Dados eletrônicos. – Porto Alegre: Bookman, 2013. Editado também como livro impresso em 2013. ISBN 978-85-65837-65-1 1. Geociências. 2. Sistema de informação geográfica. I. Longley, Paul A. CDU 528.85Longley, Paul A.. Sistemas e Ciência da Informação Geográfica (p. i). Edição do Kindle.

[16] Barata RB, de Almeida Ribeiro MCS, de Moraes JC, Flannery B (2012). Socioeconomic inequalities and vaccination coverage: results of an immunisation coverage survey in 27 Brazilian capitals, 2007–2008. J Epidemiol Community Heal.

[17] Arroyo LH, Ramos ACV, Yamamura M, Weiller TH, de Almeida Crispim J, Cartagena-Ramos D (2020). Areas with declining vaccination coverage for BCG, poliomyelitis, and MMR in Brazil (2006–2016): maps of regional heterogeneity. Cad Saude Publica..

[18] Ferreira VLR, Waldman EA, Rodrigues LC, Martineli E, Costa ÂA, Inenami M (2018). Avaliação de coberturas vacinais de crianças em uma cidade de médio porte (Brasil) utilizando registro informatizado de imunização. Cad Saude Publica.

[19] Nóvoa T d’Avila, Cordovil VR, Pantoja GM, Ribeiro MES, Cunha AC dos S, Benjamin AIM (2020). Cobertura vacinal do programa nacional de imunizações (PNI)/Vacinal coverage of the national immunization program (PNI). Brazilian J Heal Rev.

[20] Ministério da Saúde (BR), Secretaria de Vigilância em Saúde (2019). Avaliação dos indicadores de desempenho da vacinação do Programa Nacional de Imunizações e os desafios para elevar as coberturas vacinais no Brasil.

[21] Oliveira G da S, Bitencourt EL, Amaral PFF, Vaz GP, Júnior PMR, Costa SB da (2020). Cobertura vacinal: uma análise comparativa entre os estados da Região Norte do Brasil. Rev Patol do Tocantins.

[22] Queiroz LLC, Monteiro SG, Mochel EG, Veras MA de SM, de Sousa FGM, Bezerra ML de M (2013). Cobertura vacinal do esquema básico para o primeiro ano de vida nas capitais do Nordeste brasileiro. Cad Saude Publica.

[23] Santos TG dos, Jesus APM de, Oliveira FKF, Souza AB de L, Hora AB, Fraga ASB (2021). Cobertura Vacinal de rotina em crianças menores de um ano em Sergipe. Enferm Rev.

[24] Khan J, Shil A, Prakash R (2018). Exploring the spatial heterogeneity in different doses of vaccination coverage in India. PLoS ONE.

[25] Vyas P, Kim D, Adams A (2019). Understanding Spatial and Contextual Factors Influencing Intraregional Differences in Child Vaccination Coverage in Bangladesh. Asia-Pacific J Public Heal.

[26] Joy TM, George S, Paul N, Renjini BA, Rakesh PS, Sreedevi A (2019). Assessment of vaccine coverage and associated factors among children in urban agglomerations of Kochi, Kerala. India J Fam Med Prim Care.

[27] Yourkavitch J, Burgert-Brucker C, Assaf S, Delgado S (2018). Using geographical analysis to identify child health inequality in sub-Saharan Africa. PLoS ONE.

[28] Brearley L, Eggers R, Steinglass R, Vandelaer J (2013). Applying an equity lens in the Decade of Vaccines. Vaccine.

[29] Martins L, Cunha N, Baebieri C, Pamplona Y, Loureiro N, Olinda R (2020). Spatial analysis of vaccination coverage in Paraíba in 2016. Eur J Public Health.

[30] Barbieri CLA, et al. Imunização e Cobertura Vacinal: Passado, Presente e Futuro. São Paulo; Ed. Leopoldianum; 2021.

[31] Conselho Nacional de Secretarios de Saúde (CONASS). Indicadores universais do rol de diretrizes, objetivos, metas e indicadores: 2013–2015. Guia de apoio à gestão estadual do SUS. 2016. Brasília; 2016. http://www.conass.org.br/guiainformacao/category/indicadores-universais-dorolde-diretrizes-objetivos-metas-e-indicadores-2013-2015-coap/ .

